# Inhibition of *Klebsiella* β-Lactamases (SHV-1 and KPC-2) by Avibactam: A Structural Study

**DOI:** 10.1371/journal.pone.0136813

**Published:** 2015-09-04

**Authors:** Nikhil P. Krishnan, Nhu Q. Nguyen, Krisztina M. Papp-Wallace, Robert A. Bonomo, Focco van den Akker

**Affiliations:** 1 Department of Biochemistry, Case Western Reserve University, 10900 Euclid Ave., Cleveland, OH, 44106, United States of America; 2 Research Service, Louis Stokes Cleveland Department of Veterans Affairs Medical Center, 10701 East Boulevard, Cleveland, OH, 44106, United States of America; 3 Department of Medicine, Case Western Reserve University, Cleveland, OH, United States of America; 4 Department of Pharmacology, Case Western Reserve University, Cleveland, OH, United States of America; 5 Department of Molecular Biology and Microbiology, Case Western Reserve University, Cleveland, OH, United States of America; Weizmann Institute of Science, ISRAEL

## Abstract

β-Lactamase inhibition is an important clinical strategy in overcoming β-lactamase-mediated resistance to β-lactam antibiotics in Gram negative bacteria. A new β-lactamase inhibitor, avibactam, is entering the clinical arena and promising to be a major step forward in our antibiotic armamentarium. Avibactam has remarkable broad-spectrum activity in being able to inhibit classes A, C, and some class D β-lactamases. We present here structural investigations into class A β-lactamase inhibition by avibactam as we report the crystal structures of SHV-1, the chromosomal penicillinase of *Klebsiella pneumoniae*, and KPC-2, an acquired carbapenemase found in the same pathogen, complexed with avibactam. The 1.80 Å KPC-2 and 1.42 Å resolution SHV-1 β-lactamase avibactam complex structures reveal avibactam covalently bonded to the catalytic S70 residue. Analysis of the interactions and chair-shaped conformation of avibactam bound to the active sites of KPC-2 and SHV-1 provides structural insights into recently laboratory-generated amino acid substitutions that result in resistance to avibactam in KPC-2 and SHV-1. Furthermore, we observed several important differences in the interactions with amino acid residues, in particular that avibactam forms hydrogen bonds to S130 in KPC-2 but not in SHV-1, that can possibly explain some of the different kinetic constants of inhibition. Our observations provide a possible reason for the ability of KPC-2 β-lactamase to slowly desulfate avibactam with a potential role for the stereochemistry around the N1 atom of avibactam and/or the presence of an active site water molecule that could aid in avibactam desulfation, an unexpected consequence of novel inhibition chemistry.

## Introduction

**Fig 1:**
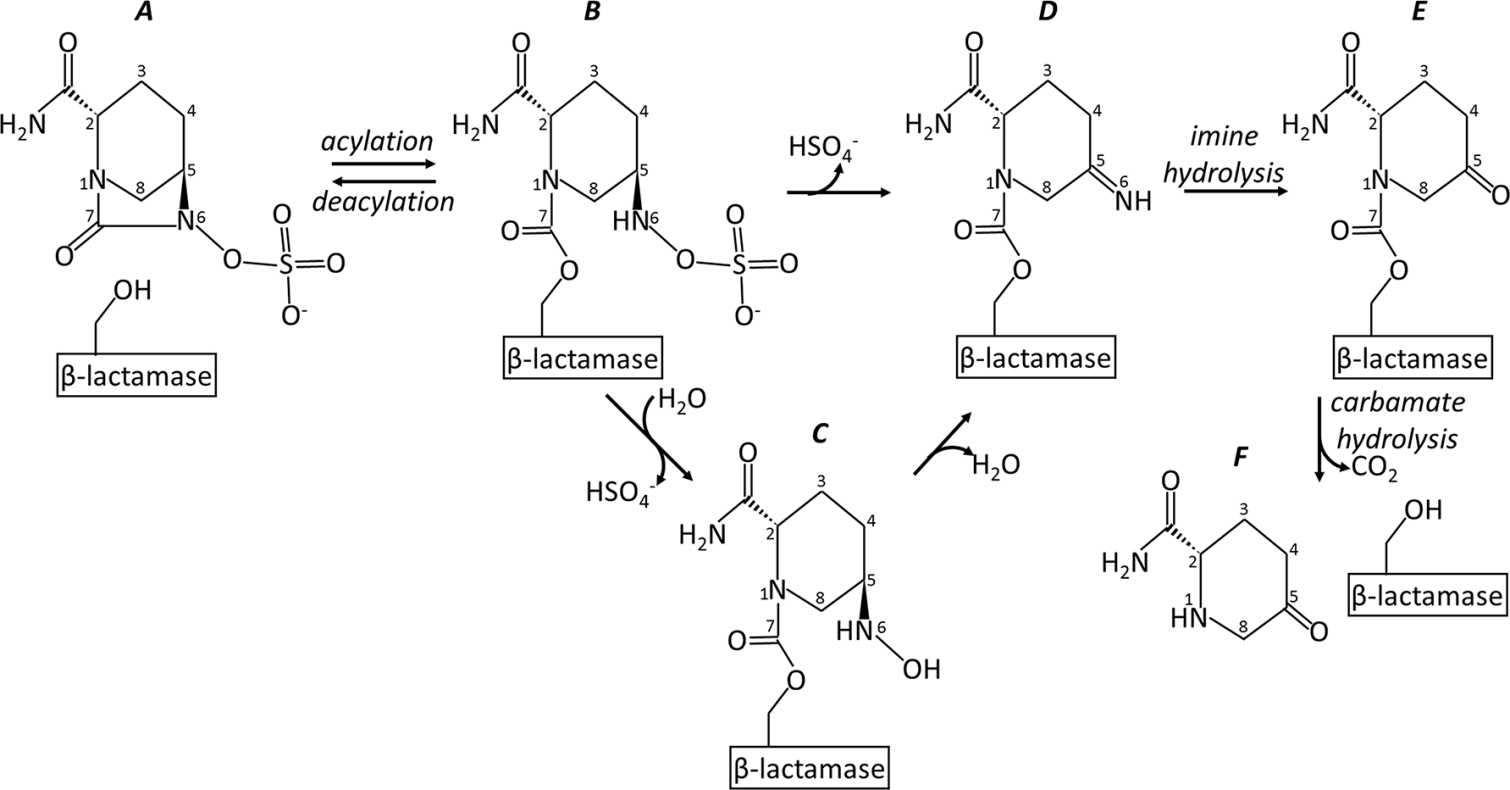
Schematic diagram of avibactam inhibition and deacylation pathways of KPC-2.

Bacterial β-lactamase enzymes are a major contributor to antibiotic resistance by hydrolyzing β-lactam antibiotics [1]. A proven strategy to overcome such a resistance mechanism is to combine a β-lactam antibiotic with a β-lactamase inhibitor [2]. This led to the clinical introduction of three inhibitors: tazobactam, sulbactam, and clavulanic acid. The plethora of new enzymes and the emergence of inhibitor resistant β-lactamases in the clinic spurred the development of new β-lactamase inhibitors. Most recently, ceftazidime-avibactam, the latter being a novel bridged diazabicyclooctane (DBO) non-β-lactam β-lactamase inhibitor (NXL104, Fig 1), was approved by the Food and Drug Administration (FDA) (www.fda.gov). Unlike the first 3 clinical inhibitors, avibactam does not contain a β-lactam core and also does not contain a carboxyl moiety. Furthermore, avibactam inhibits serine β-lactamases via a reversible mechanism which differentiates it from the currently available inhibitors [3].

Despite these differences, avibactam shares mechanistic similarities with the other inhibitors; these properties include utilization of the oxyanion hole to accommodate the carbonyl oxygen for stabilizing the transition state, carbonyl carbon acylation of S70, and bond breakage between the carbonyl carbon and the nitrogen of avibactam (Fig 1). In contrast to other inhibitors, avibactam is deacylated and released from the enzyme in a reversible manner (i.e. recyclization) via ring closure regenerating the intact avibactam that can now again acylate other, or the same, β-lactamases [3]. In contrast, the other 3 inhibitors either form different inhibitory intermediates [4–6] and/or can form a covalent irreversible adduct [6], and can deacylate resulting in an inactive inhibitor fragment [2].

Avibactam, which is used in combination with the β-lactam antibiotic ceftazidime, possesses a relatively broad-spectrum inhibition activity as it can inhibit class A, C, and some class D β-lactamases [7] including extended-spectrum β-lactamases such as SHV variants [8]. Unlike the currently available inhibitors avibactam possesses the unique ability to also inhibit KPC-2 [7, 9–11]. KPC-2 hydrolyzes clavulanic acid, is phenotypically resistant to inhibitors and bacteria expressing this class A β-lactamase represent a major global health threat. Infections caused by *Klebsiella pneumoniae* and *Escherichia coli* harboring KPCs are notoriously difficult to treat [12] with numerous outbreaks reported [13–15]. The global spread of KPC is a cause for serious alarm, in particular since patients infected with bacteria expressing KPC exhibit a high mortality rate due to limited treatment options [16].

The crystal structure of apo KPC-2 [17] and KPC-2 with inhibitors (3-nitrophenyl boronic acid and a penam sulfone, PSR-3–226) were previously determined [18]. The inhibition of KPC-2 by avibactam differs from the inhibition of other β-lactamases; avibactam, in addition to reversible deacylation, undergoes a slow desulfation [7]. Desulfation results in a deacylated avibactam fragment that cannot reacylate leading to degradation of this inhibitor instead of the reversible inhibition observed for the other β-lactamases. One could hypothesize that this process potentially hampers the efficacy of avibactam against KPC-2. On a mechanistic basis, considering the two pathways (recyclization vs. desulfation), recyclization is favored [7]. Based upon kinetics, the t_1/2_ of recyclization is ~ 82 min for KPC-2 and, using mass spectrometry, a t_1/2_ of ~7 hr for KPC-2 exists for desulfation-deacylation [7]. Furthermore, a recent study indicated that certain laboratory-generated KPC-2 variants are resistant to avibactam *in vitro* [19] which could affect the future efficacy of avibactam if these variants were to develop in the clinical population. In light of the medical importance of avibactam inhibition of KPC-2 and the different possible release pathways (recyclization and desulfation-deacylation) of the avibactam bound to this carbapenemase, we present here the structural investigation of KPC-2 inhibition by avibactam. In addition, we have also determined the structure of avibactam bound to SHV-1. This latter enzyme is also found in *K*. *pneumoniae*, which is often responsible for hospital-acquired infections with SHV variants demonstrating an ESBL phenotype. A recent study also indicated that similar engineered and naturally occurring substitutions in SHV-1 could also render this enzyme avibactam-resistant [20]. Here, we present a comparative structural investigation into avibactam binding modes, avibactam resistance mutations, and differential avibactam degradation by KPC-2 and SHV-1 including comparisons with previously determined avibactam complexes involving CTX-M-15, AmpC, OXA-10, OXA-24, OXA-48, and BlaC [21–24].

## Materials and Methods

The SHV-1 and KPC-2 β-lactamases were expressed and crystallized as described previously [18, 25]. As before, we used the C-terminally truncated KPC-2 β-lactamase in which the last 4 residues were removed to facilitate improved crystallization [18, 26]. KPC-2 was crystallized using vapour diffusion using a sitting drop tray with a well solution comprised of 20% PEG 6000, 100 mM potassium thiocyanate, and 100 mM citrate pH 4.0; SHV-1 was crystallized using 25% PEG 6000, 100mM Tris pH 7.5, and 0.56mM Cymal-6. A crystal of SHV-1 was soaked with 50mM avibactam (AstraZeneca, Waltham, Massachusetts, USA) in mother liquor for 40 min prior to transfer to perfluoropolyether for cryo-protection before flash freezing the crystal in liquid nitrogen. KPC-2 crystals were soaked for 8 min with 5mM avibactam in mother liquor (25% PEG 6000, 100mM citrate pH 5.0) and 20% ethylene glycol prior to flash freezing. Data was collected at the Stanford Synchrotron Radiation Lightsource and processed using HKL2000[27] resulting in 1.42Å and 1.8Å datasets for SHV-1 and KPC-2, respectively (data collection statistics are shown in Table 1). Starting protein coordinates for SHV-1 and KPC-2 were PDB identifiers 2H5S and 3RXW, respectively; the program MOLREP was used for molecular replacement [28]. The structure was refined using REFMAC and COOT[29, 30]. Initial refinement and subsequent density inspection indicated the presence of a covalently bound avibactam in both the SHV-1 and KPC-2 active sites. The program PRODRG[31] was used to obtain the topology and refinement parameter files for avibactam for subsequent inclusion of the ligand in refinement. The program PROCHECK was used for structure validation [32]. Final refinement statistics are listed in Table 1. The coordinates and structure factors for the KPC-2 and SHV-1 avibactam complexes were deposited with the Protein Data Bank (PDB identifiers 4ZAM and 4ZBE, respectively).

**Table 1:** Data collection and refinement statistics for SHV-1 and KPC-2 soaked with avibactam.

*Data collection*	*SHV-1*	*KPC-2*
Space group	*P*2_1_2_1_2_1_	*P*2_1_2_1_2_1_
Unit cell dimensions (Å)	49.40 55.30 85.37	51.32 66.70 73.21
Wavelength (Å)	1.1271	1.1272
Resolution (Å)	50–1.42	50–1.80
Redundancy	3.5	2.9
Unique reflections	44,246	22,206
<I>/<σ(I)>	27.0 (3.6)	15.6 (1.9)
Rmerge (%)	3.7 (33.8)	5.3 (44.4)
Completeness (%)	98.3 (89.3)	93.2 (88.2)
*Refinement*		
Resolution range (Å)	37–1.42	37–1.8
R-factor (%)	16.6%	15.6%
R_free_ (%)	19.3%	21.9%
RMSD deviation from ideality		
Bond lengths (Å)	0.013	0.018
Angles (deg)	1.75	1.77

## Results and Discussion

**Fig 2:**
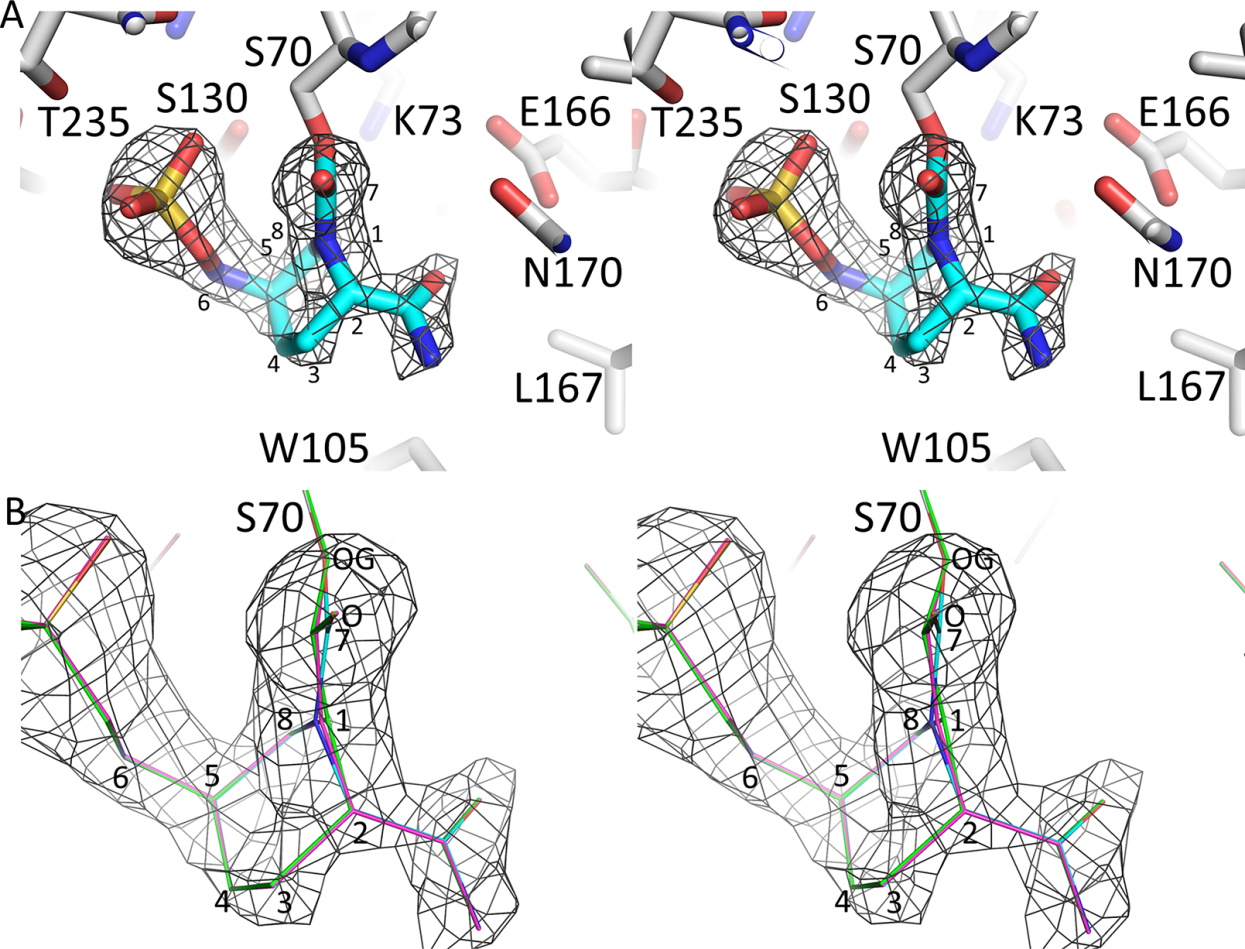
Electron density of avibactam bound to the active site of KPC-2. A, |Fo|-|Fc| electron density difference density is depicted for the ligand (contoured at 3.25σ). The covalently-bound avibactam is shown in blue stick model. Prior to the map calculation, the model with the avibactam ligand removed has been subjected to 10 cycles of Refmac crystallographic refinement. B, close-up view of the electron density around the N1 atom of avibactam with 3 different models of avibactam: avibactam refined with the N1 in the *S* enantiomer conformation (same colors as in A), avibactam refined with the N1 in the planar conformation (all atoms in magenta), and avibactam refined with the N1 in the *R* enantiomer conformation (all atoms in green). By changing the refined chirality of the N1 atom, the resulting model yields a different position for the C7 atom of avibactam thus distorting the planarity of the adjacent carbonyl moiety (involving avibactam atoms N1, C7, O, and S70 atom OG). As a measure of this carbonyl planarity, the OG atom distance from the plane defined by avibactam atoms N1, C7, and O is 0.15, 0.56, and 0.86 Å for the *S*, planar, and *R* enantiomer conformation of N1, respectively.

**Fig 3:**
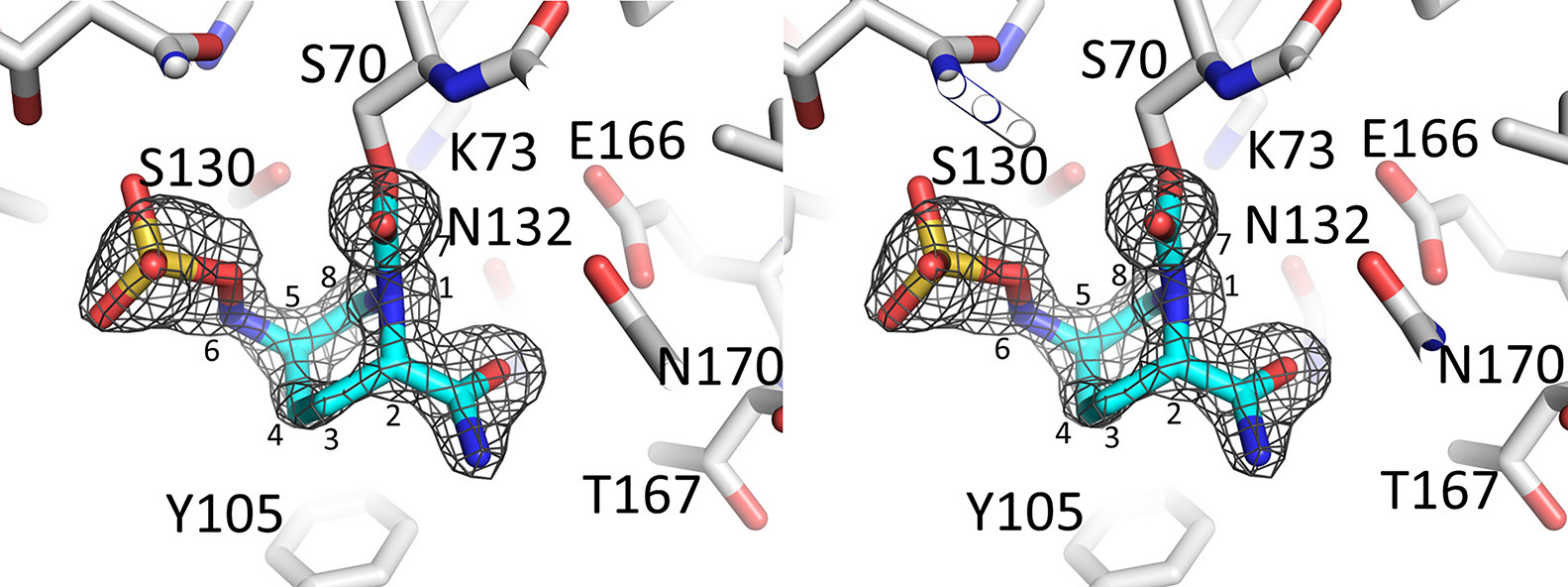
Electron density of avibactam bound to the active site of SHV-1. |Fo|-|Fc| electron density difference density is depicted for the ligand (contoured at 3.25σ). The covalently-bound avibactam is shown in blue stick model. The map was calculated similar to that in Fig 2.

**Fig 4:**
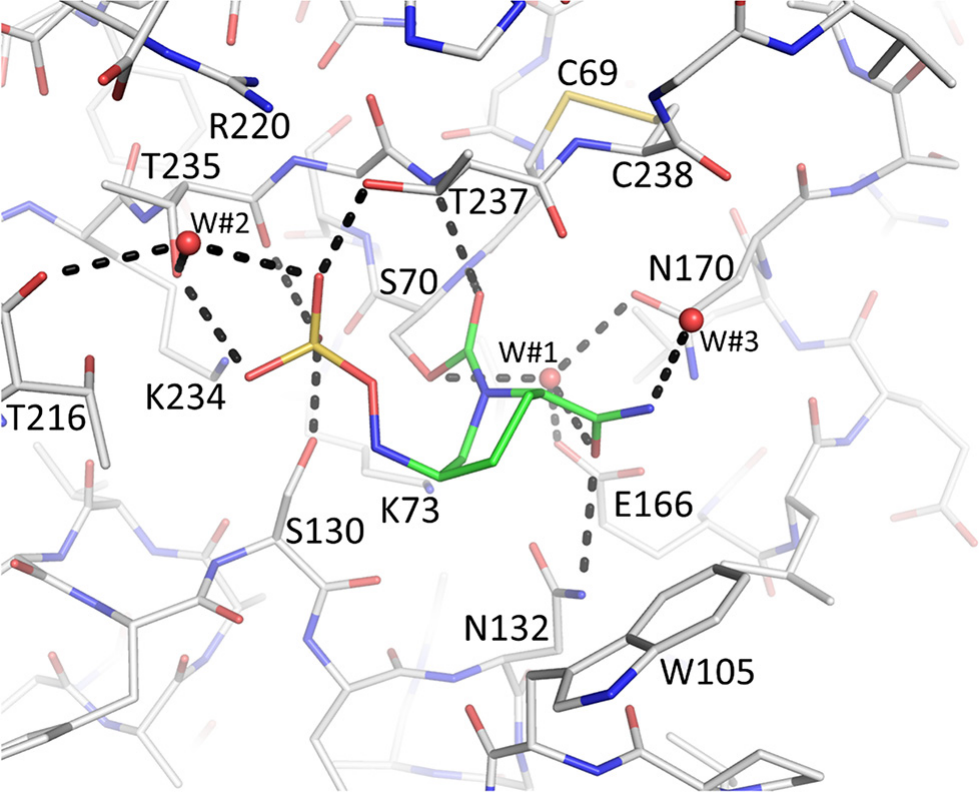
Interactions of avibactam in the active site of KPC-2. Avibactam is shown with green carbon atoms. Hydrogen bonds are depicted as dashed lines (cut-off distance is 3.2Å). The deacylation water is present (labeled W#1). Additional waters are labeled W#2–3.

We found avibactam covalently bonded to S70 in the active site of KPC-2 and SHV-1 (Figs 2 and 3). Omit electron density is strong for all avibactam moieties including the sulfate and amide moieties. The 6-membered ring of avibactam adopts a “chair conformation” in both structures. When bound to KPC-2, avibactam makes hydrogen bonds via its carbonyl oxygen in the oxyanion hole (comprised of backbone nitrogen atoms of S70 and T237), via the amide moiety with N132, and via its sulfate moiety with residues S130, T237, T235, and the main chain oxygen of T235 (Fig 4). Residue W105, located 4.4 Å away is providing modest van der Waals interactions with the C4 atom of avibactam (Figs 2 and 4). In addition, K234 is potentially making a long range hydrogen bond electrostatic interaction with the sulfate moiety of avibactam with a distance of slightly greater than 3.3Å (not shown in Fig 4 due to cut-off 3.2Å distance used).

**Fig 5:**
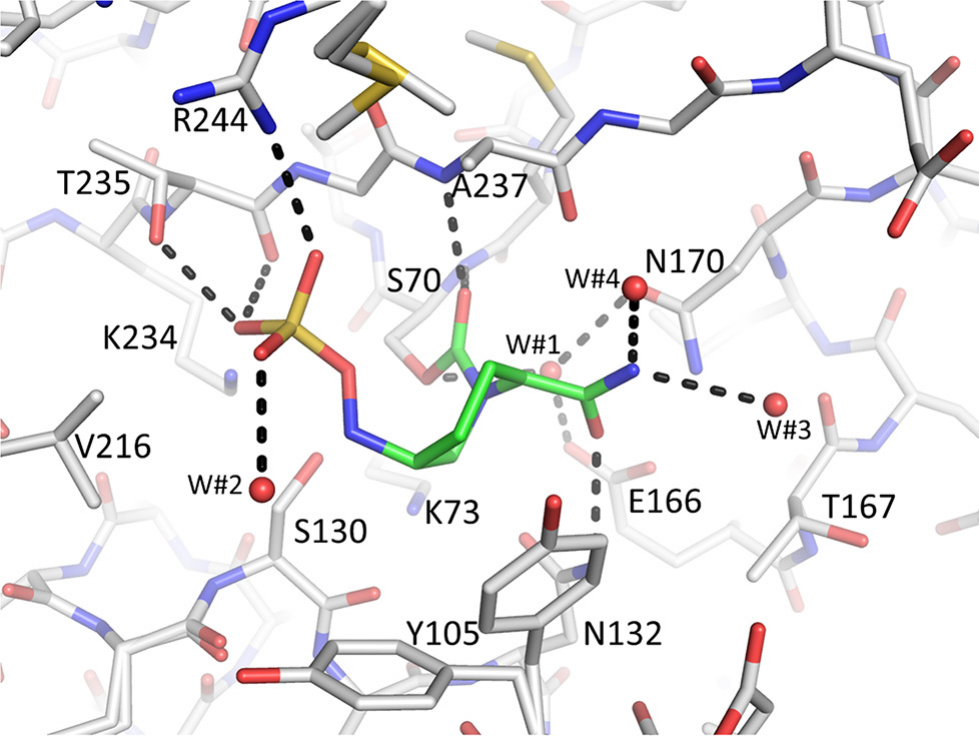
Avibactam in the active site of SHV-1. Interactions of avibactam (shown with green carbon atoms) in the active site of SHV-1. Hydrogen bonds are depicted as dashed lines. The deacylation water is present (labeled W#1). Additional waters are labeled W#2–4.

**Fig 6:**
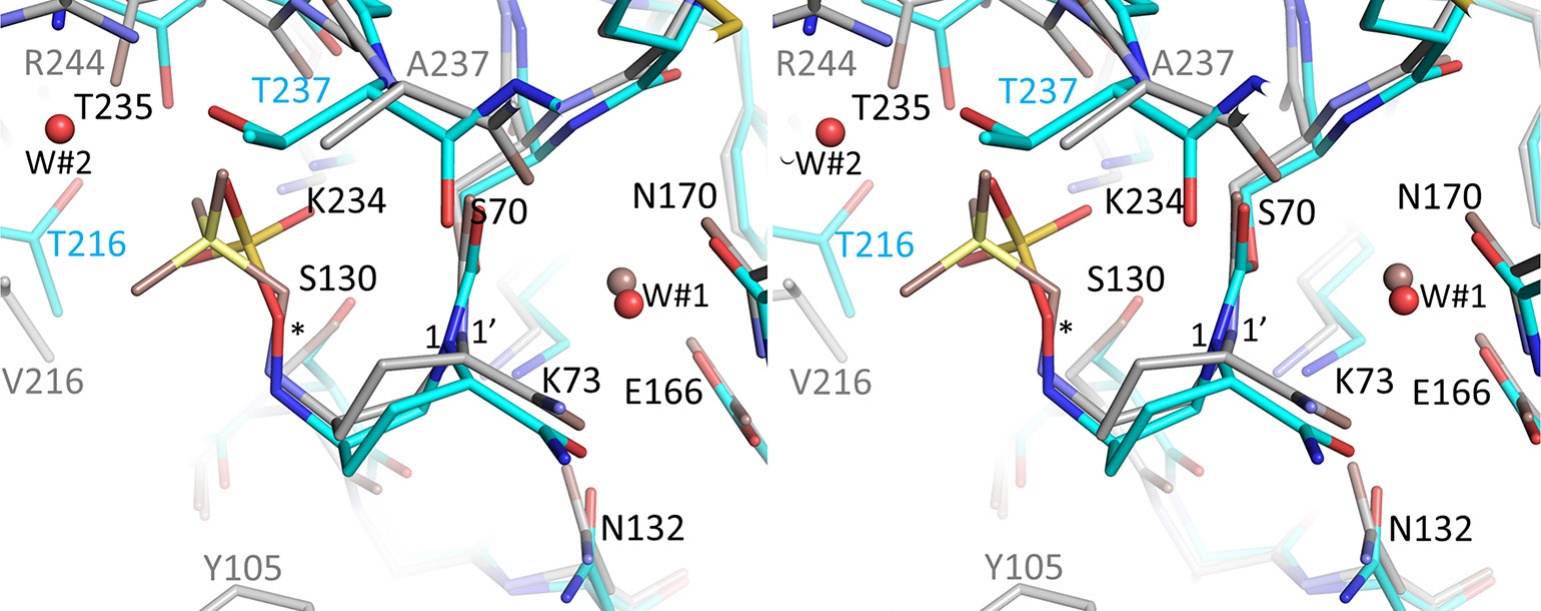
Superposition of complexes of KPC-2 and SHV-1 bound with avibactam. Superimposed are KPC-2 (cyan carbon atoms) and SHV-1 (grey carbon atoms). The nitrogen N1 is labelled ‘1’ and ‘1’” for KPC-2 and SHV-1, respectively, to indicate the differences in the chirality and direction of the lone pair electrons that nitrogen in the 2 different structures. The oxygen of the sulfate moiety of avibactam bound to KPC-2 is indicated by a ‘*’. The deacylation water, W#1, and sulfate hydrogen-bonding water in KPC-2, W#2, are indicated as well. The following active site residues were used for the superpositioning: SHV-1 residues 233–238, 68–84, 121–140, 167–172 onto the equivalent residues of KPC-2; root-mean-square-deviation is 0.51Å for 48 Cα atoms.

The structure of avibactam complexed to SHV-1 shows that this DBO inhibitor is covalently bound in a similar fashion in the active site (Figs 5 and 6) compared to KPC-2 although there as some differences. An important difference is the sulfate moiety of avibactam makes an arginine-mediated salt bridge interaction in SHV-1 involving R244, while in KPC-2 this is absent. An additional contributing difference is that T237 in KPC-2 is more bulky than the corresponding A237 in SHV-1 and likely forces a shift in the avibactam sulfate moiety. In addition to the difference in the sulfate binding region, another variation is that Y105 in SHV-1 is disordered and occupies two conformations (Figs 3 & 5) whereas its corresponding residue in KPC-2, W105, occupies a single conformation (Figs 2 & 4).

**Fig 7:**
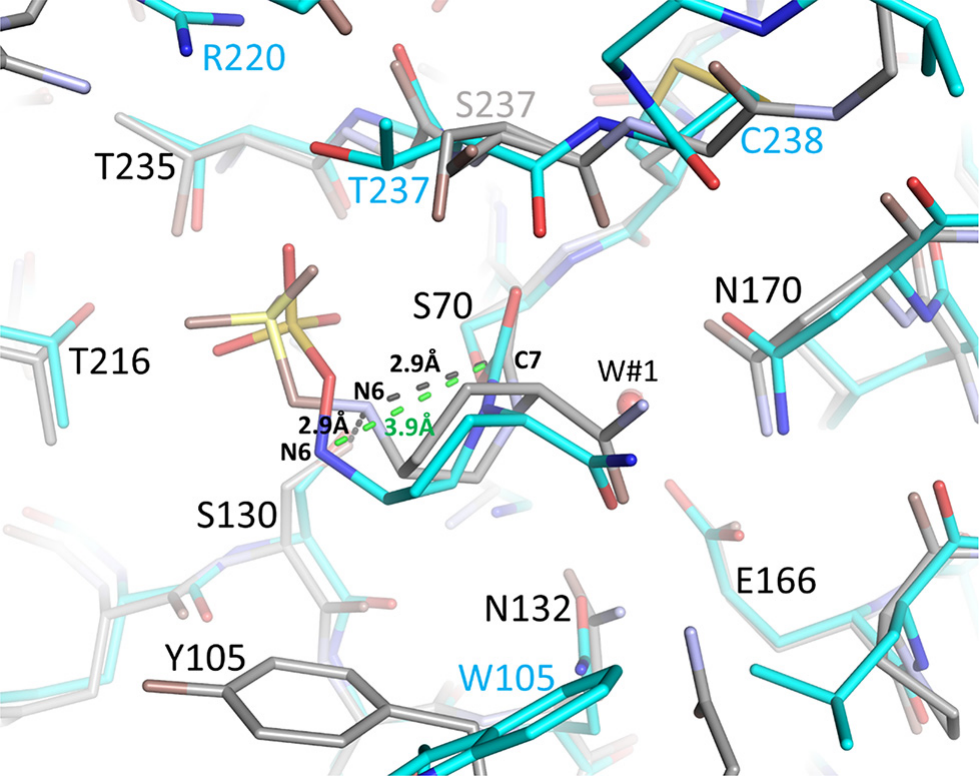
Superposition of complexes of KPC-2 and CTX-M-15 bound with avibactam. Superimposed are CTX-M-15 (grey carbons atoms) and KPC-2 (cyan carbon atoms). The deacylation water, W#1, is labelled. Key distance differences listed in Table 2 are indicated by dashed lines. These distance differences involve the avibactam N6 atom pointing towards the avibactam C7 atom in CTX-M-15 (listed in black) whereas in KPC-2 this avibactam N6 atom is pointing away thus being situated at a larger distance (listed in green). In addition, the avibactam N6 hydrogen bond with S130 in CTX-M-1 is depicted as well; this is not present in KPC-2.

Regarding the mechanism of avibactam inhibition of class A β-lactamases, the general consensus is that the catalytic S70 residue is primed for nucleophilic attack by deprotonation, but whether this deprotonation is mediated by K73 or E166, via a water molecule bridging both E166 and S70 (W#1 in Figs 4 & 5), is still debated [23, 24, 33]. The subsequent step during acylation (i.e, the protonation of the N6 atom of avibactam) is postulated to be mediated by S130 aided by K73 [23, 24, 33]. Avibactam deacylation likely involves S130 and is assisted by residue K73 which is deeper in the active site yet in close proximity to S130 [24]. The observed binding mode of avibactam bound to both KPC-2 and SHV-1 is similar to that when it is bound to CTX-M-15 [23, 24] (Figs 6 and 7) which is in agreement with these kinetic, mutagenesis, and molecular modeling studies of avibactam inhibition in homologous class A β-lactamases [19, 20].

The observed presence of the covalent bond involving S70 as well as the hydrogen bonding network between S130 and K73, and K234 and S70 are all conserved in CTX-M-15, SHV-1, and KPC-2 although the distance between S130 and K73 is somewhat larger in KPC-2 (3.35 Å) compared to the 2.8 Å and 3.1 Å distances observed in CTX-M-15 and SHV-1, respectively. The “deacylation” water that is utilized by class A β-lactamases is present in both the SHV-1 and KPC-2 avibactam complexes (W#1 in Figs 4 & 5). This water is interacting with E166 and N170 and is normally primed to deacylate substrates or mechanism-based β-lactamase inhibitors. In some β-lactamase inhibitor structures, the water is moved. However, this water does not seem to play a role in avibactam deacylation. Likely, the carbamoyl bond of avibactam with S70 is more resistant to deacylation compared to ester linkage utilized by β-lactam substrates and most inhibitors [3, 24]. In addition, the presence of the negatively charged sulfate moiety in close proximity to the carbonyl moiety could also affect the reactivity of avibactam towards the deacylation water as was speculated to be one of the reasons for the increased *trans*-enamine stability of the SA2–13 inhibitor; this inhibitor also has a negatively charged moiety occupying a similar position [25].

A very notable difference between the binding mode of avibactam bound to CTX-M-15 compared to SHV-1 and KPC-2 is the way the N6 atom of avibactam is oriented (Figs 6 and 7 and Table 2). In CTX-M-15, this N6 atom is in close proximity to the C7 atom of avibactam (2.9 Å) and also makes a hydrogen bond with S130 (2.9 Å) such that this binding mode is primed for re-cyclization of avibactam, *via* reformation of the N6-C7 bond, aided by S130 as the proposed general base to abstract the proton from N6 [23, 24]. In contrast, in SHV-1 and KPC-2, the avibactam N6 atom is not pointing towards its C7 atom as the distance between these atoms is greater than 3.8 Å in both structures (Figs 6 and 7). Also, the N6 atom of avibactam is situated further from the hydroxyl atom of S130 (i.e. 3.3 Å in SHV-1 and 3.7 Å when bound to KPC-2). Overall, these observed structural differences could, in part, be the basis of the roughly 2-fold slower avibactam deacylation/recyclization rate for KPC-2 compared to CTX-M-15 [7], as the latter conformation of avibactam seemed better primed for deacylation via recyclization (as discussed above).

**Table 2:** Comparison of avibactam inhibition kinetics and avibactam active site interaction.

	% acyl after 24 hr	Deacyl t_1/2_ (min)	*K*_d_ (μM)	ChiralityN1^a^	Direction N6^b^	# H-bonds sulfate^c^	N6 H-bonds (<3.3 Å)^d^	O H-bonds^e^	H_2_O^f^	R/K^g^
**KPC-2**[7],this study	10	82	0.011	*S*	away	4	n	n	y	n
***P*.*a*. AmpC** [7],[23], ([22])	71	6	0.66	*R*/planar (*R*)	Away (sideways)	1 (3)	n (y)	n (y)	y (y)	y (n)
**OXA-10**[7],[24]	91	>7200	<0.15	*R*	away	4	y	n	n	y
**OXA-48** [7],[23]([24])	97	1000	0.009	planar	away	3 (3–6)	y (y)	n (n)	n (n)	y
**CTX-M-15** [7],[23]([24])	100	40	0.002	*R*	inwards	2 (3)	y (y)	n (n)	n (y)	n (n)
**SHV-1**[23],this study	100			*R*	away	3	n	n	n	y
***M*.*t*. BlaC**[21]	98		>500	planar	away	3	y	y	y	n
**OXA-24**[22]		1823	0.12	planar	away	4	y	n	y	y

^a^the chirality of avibactam tertiary N1 atom

^b^the direction of N6 inwards towards the carbonyl carbon or away towards the solvent

^c^the number of hydrogen bonds made with the oxygen atoms of the sulfate

^d^hydrogen bonds formed with the nitrogen N6 of avibactam

^e^hydrogen bond formed with the sulfate oxygen adjacent the N6 atom

^f^presence of an active site water hydrogen bonding to an oxygen atom of the sulfate and positioned above the plane of the 3 oxygen atoms to potentially initiate a nucleophilic attack (as in Fig 1)

^g^sulfate moiety is within hydrogen bonding distance of a positively charged side chain (Arg (R) or Lys (K)).

The KPC-2:avibactam structure aids in explaining the structural basis of engineered substitutions that were shown to lead to resistance to this inhibitor against KPC-2 [19]. The substitutions studied include, with the MICs for ampicillin/avibactam listed in parentheses, wild-type KPC-2 without substitutions (2–8 mg/L), S130G (512 mg/L), K234R (64 mg/L), and R220M (32 mg/L). Of these 3 substitutions, the S130G variant leads to the largest increase in resistance likely due to its role in multiple aspects of avibactam inhibition. We will now discuss the importance of each.

### S130G

Biochemical evaluation of the S130G variant in KPC-2 showed a 100-fold decrease in affinity, an 18,000-fold decrease in acylation (*k*_*2*_/*K*), and a 7,000-fold increase in ratio of inhibitor:enzyme needed to yield a 90% inhibition after 24hr [19]. These differences in inactivation kinetics for S130G KPC-2 could potentially have a structural/mechanistic basis due to the following three reasons: 1) the S130 directly hydrogen bonds to the acyl avibactam complex as observed in the KPC-2 structure (Fig 4); 2) S130 has a major role in avibactam recognition in the Michaelis-Menten/pre-acylation complex (similar to an inhibitor SHV-1 complex as observed previously [34]); 3) S130 is the postulated general acid that donates a hydrogen to the N6 nitrogen of avibactam [24]. The turnover number (partition ratio) is drastically increased for the S130G substitution in KPC-2, whereas this value does not change for the same substitution in SHV-1. A potential factor in this kinetic difference could be due to the possible reliance of KPC-2 on S130 *via* the hydrogen bond in KPC-2 with one of the sulfate oxygens whereas a hydrogen bond is not observed in SHV-1 (Figs 4 and 5).

### K234R

The second residue that impacts avibactam inhibition in KPC-2 is K234R. This change (K →R) has an intermediate effect on ampicillin/avibactam resistance; this side chain is hydrogen bonding to S130 in the KPC-2:avibactam structure. Therefore, the K234R substitution will likely affect the position of S130 (with the above listed potential consequences) and/or influence the recognition of the sulfate/carboxylate moiety of the inhibitor in the Michaelis-Menten complex as previously proposed for the equivalent mutation in SHV-1 [34, 35]. Note that the K234R not only results in resistance to inhibition by avibactam, but also maintains catalytic activity against ampicillin [19] which is unusual for inhibitor-resistance variants in general [2].

### R220M

R220M is the third amino acid substitution in KPC-2 that has a modest effect on MIC (32 mg/L) compared to the other two changes. This is anticipated since this residue is not within hydrogen bonding distance of avibactam in the acyl complex structure, it is positioned more than 4 Å from the sulfate moiety, but could still have a significant electrostatic interaction with the negatively charged avibactam group (Fig 4). The resistance phenotype of the R220M variant can possibly be explained by invoking the following arguments: 1) by losing the potential electrostatic interaction with the sulfate moiety, the R →M substitution directly effects the formation of both the Michaelis-Menten/preacylation state and acyl-enzyme intermediates (Fig 4); 2) by potentially affecting the orientation of T237, the R →M change disrupts the two hydrogen bonds R220 makes with T237. In turn, T237 is oriented in the avibactam complex such that it makes a hydrogen bond with the sulfate moiety of avibactam (Fig 4); this T237 avibactam interaction might be disrupted by the R220M substitution via loss of the two hydrogen bonds. Additionally, the R220M change could affect the position of water W#2 which interacts with the sulfate moiety of avibactam (Fig 4).

In addition to KPC-2, a similar study regarding avibactam resistance substitutions was carried out with SHV-1 β-lactamase [20]. This latter study showed that the S130G and K234R both conferred increased resistance to avibactam/ampicillin in SHV-1. Both substitutions increased the MIC to 256 mg/L compared to 1 mg/ml for wild type SHV-1 [20]. In the SHV-1:avibactam complex, residue S130 is not making a strong hydrogen with avibactam, this amino acid is located at a distance of greater than 3.3 Å from the sulfate moiety. Nevertheless, kinetic analysis shows that the avibactam acylation rate (*k*_*2*_/*K*) is drastically decreased by 45,000 fold and the apparent *K*_*i*_ is more than 450,000 fold greater compared to wild type SHV-1 [20]. These results show a similar trend as in the S130G KPC-2 although the effect on apparent *K*_*i*_ in SHV-1 is much larger than in KPC-2. Unlike KPC-2, the ratio of inhibitor:enzyme needed to yield a 90% inhibition after 24 hr did not change by the S130G alteration and remained 1, compared to wild-type SVH-1 compared to 7,000 for KPC-2 [19, 20].

### Possible structural basis for KPC-2-mediated avibactam desulfation

KPC-2, and to a lesser degree *Pseudomonas aeruginosa* AmpC, were found to be somewhat different compared to five other β-lactamases regarding the proportion of acylated β-lactamase that remained after 24 hr. The other tested β-lactamases TEM-1, CTX-M-15, *E*. *cloacae* AmpC, OXA-10, and OXA48 remained more than 90% acylated after 24 hr [7]. KPC-2 and *P*. *aeruginosa* AmpC were 100% acylated by avibactam after 300s, however after 24 hr incubation at 37°C this decreased to 10% and 71%, respectively [7]. For KPC-2, incubation at room temperature for 24 hr did not reveal such a decrease for avibactam [19] suggesting a temperature dependence of this deacylation by desulfation/hydrolysis. Note that the 24 hr incubation experiment involves numerous acylation, reversible deacylation, and reacylation occurrences of the same avibactam molecules as the deacylation by recyclization half-life was generally in the order of tens or hundreds of minutes.

Investigating the manner in which KPC-2 undergoes desulfation-hydrolytic deacylation *via* detailed kinetic and mass spectroscopy analysis concluded that avibactam loses its sulfate moiety either directly or *via* a water-mediated step[7] and the resulting avibactam fragment cannot reacylate KPC-2 (see Fig 1). Although not investigated, *P*. *aeruginosa* AmpC reduction in amount of acylated β-lactamase could potentially also be caused by such a desulfation mechanism. Alternatively this could also be due to its higher *K*_d_ for avibactam binding compared to the other enzymes tested.

In order to understand the mechanistic basis for desulfation reaction in KPC-2, we compare our avibactam structure to previously determined complexes. We chose to examine OXA-10 [24], OXA-48 [22, 24], CTX-M-15 [23], *P*. *aeruginosa* AmpC [23], SHV-1, and BlaC of *M*. *tuberculosis* [21]. We chose BlaC and SHV-1 as they did not show any change in proportion of acyl enzyme after 24 hr [20, 21] although these latter experiments were carried out at room temperature which might not be conducive to avibactam sulfate fragmentation (as noted above). OXA-24 enzyme was included even though a 24hr avibactam acylation experiment was not done although other kinetic inhibition experiments were carried out for this enzyme [22].

Super positioning of avibactam complexes of KPC-2 onto SHV-1 (Fig 6) and KPC-2 onto CTX-M-15 (Fig 7) suggest that avibactam adopts a similar overall binding mode despite active site differences in these class A β-lactamases. Examining other avibactam β-lactamase structures, belonging to either class C or D, also indicates that avibactam is bound in a similar fashion (data not shown). Since KPC-2 is the one exception of being able to hydrolyze the avibactam sulfate moiety [7], we more carefully compared the active sites of the different β-lactamases in order to find a possible structural explanation. Some notable differences became evident.

One important aspect that differs for KPC-2 compared to the other β-lactamases is the chirality of the N1 atom in the region of the sulfate moiety (Table 2 and Figs 2 and 6). The chirality of N1 of avibactam is likely *S* in KPC-2 (Fig 2B) although the 1.8Å resolution of this structure is not sufficient to assign this chirality with 100% confidence; in the other β-lactamases, it is either *R* or planar (Table 2). Nitrogen inversion has an energy barrier typically around 16–35 kJ/mol[36] and such inversion can occur at room temperature. A possible explanation for the KPC-2 N-inversion chirality difference could be that the active site of KPC-2 was observed to be more shallow compared to other class A β-lactamases[17].

In addition to the N1 avibactam chirality, other active site differences could potentially be responsible for the desulfation differences. If we hypothesize that *P*. *aeruginosa* AmpC also has the ability to desulfate avibactam, albeit to a lesser degree than KPC-2, the following important active site interactions could play a role as they tend to be only observed in KPC-2 and *P*. *aeruginosa* AmpC (Table 2, ignoring the last 3 β-lactamases as their desulfation experiment were not carried out 37°C): a) lack of hydrogen bonds with the N6 atom, and b) the presence of a water molecule (W#2 in Fig 4) that could potentially serve to desulfate avibactam (see also Fig 1). Among the above listed potential reasons, the one that contributes the most to the desulfation ability of KPC-2 remains unclear and additional studies are needed to narrow down these possibilities.

In summary, the crystal structures of KPC-2 and SHV-1 β-lactamase in complex with avibactam reveal interesting similarities and differences compared with previously determined avibactam complexes. Most notably, the chair conformation of avibactam is conserved yet the orientation of the N6 atom is different in both KPC-2 and SHV-1 compared to the other Class A β-lactamase complex previously determined. To illustrate in the CTX-M-15 structure, the avibactam N6 orientation is more primed for re-cyclization. Detailed structural comparisons also yielded possible reasons (e.g., the N1 avibactam chirality and presence of a water molecule) for the ability of KPC-2 to desulfate avibactam whereas the other β-lactamases do not seem to desulfate avibactam.
